# Regulatory T Cells: An Emerging Player in Radiation-Induced Lung Injury

**DOI:** 10.3389/fimmu.2020.01769

**Published:** 2020-08-04

**Authors:** Tiantian Guo, Liqing Zou, Jianjiao Ni, Yue Zhou, Luxi Ye, Xi Yang, Zhengfei Zhu

**Affiliations:** ^1^Department of Radiation Oncology, Fudan University Shanghai Cancer Center, Shanghai, China; ^2^Department of Oncology, Shanghai Medical College, Fudan University, Shanghai, China

**Keywords:** Treg, radiotherapy, lung, pneumonitis, fibrosis

## Abstract

Regulatory T cells (Tregs), which have long been recognized as essential regulators of both inflammation and autoimmunity, also impede effective antitumor immune response due to their immunosuppressive properties. Combined radiotherapy and immunotherapeutic interventions focusing on the removal of Tregs have recently garnered interest as a promising strategy to reverse immunosuppression. Meanwhile, Tregs are emerging as a key player in the pathogenesis of radiation-induced lung injury (RILI), a frequent and potentially life-threatening complication of thoracic radiotherapy. Recognition of the critical role of Tregs in RILI raises the important question of whether radiotherapy combined with Treg-targeting immunotherapy offers any beneficial effects in the protection of normal lung tissue. This present review focuses on the contributions of Tregs to RILI, with particular emphasis on the suspected differential role of Tregs in the pneumonitic phase and fibrotic phase of RILI. We also introduce recent progress on the potential mechanisms by which Tregs modulate RILI and the crosstalk among Tregs, other infiltrating T cells, fibrocytes, and resident epithelial cells driving disease pathogenesis. Finally, we discuss whether Tregs also hold promise as a potential target for immunotherapeutic interventions for RILI.

## Introduction

Radiotherapy has a well-established role in the management of thoracic neoplasms (1). However, the lung is a radiosensitive organ, and radiation-induced lung injury (RILI), consisting of radiation-induced pneumonitis (RP) and pulmonary fibrosis (RPF), severely limits the efficacy of radiotherapy and impairs the quality of life of cancer patients, making it a paramount concern for radiation oncologists (2). A variety of factors may affect the risk of RILI, such as the radiation technique used, the volume of normal lung irradiated, dose fractionation regimen, and concurrent chemotherapy (1). From published data to date, the incidence of symptomatic RP is highest for lung cancer (5–50%), followed by mediastinal lymphatics (5–10%) and breast cancer (1–5%) (3, 4); the reported incidence of RPF is in the range of 1–51% in patients receiving thoracic radiotherapy (5–7) and 70–80% in high-dose regions (8).

Regulatory T cells (Tregs), a subset of CD4^+^ T cells, have long been recognized as essential regulators of inflammation and autoimmunity (9). Recently, Tregs have emerged as a key player in the suppression of antitumor immunity (10). The combination of radiotherapy and Treg-targeting immunotherapy has garnered interest as a promising strategy to reverse immunosuppression, albeit it may also carry potential risks of inducing treatment-related toxicities (11). Meanwhile, recent studies have expanded our understanding on how Tregs modulate fibrogenesis. Intriguingly, Tregs have been shown to be both a friend and a foe of fibrosis; depending on disease stages and their interplay with other immune cells, Tregs may exert anti- or pro-fibrotic effects (12–15). Notably, growing evidence highlights the involvement of Tregs in RPF (16–23), raising the important question of whether radiotherapy combined with Treg-targeting immunotherapy offers any beneficial effects in the protection of normal lung tissue.

In this review, we summarize the current knowledge on the contributions of Tregs to RILI, with particular emphasis on the suspected differential roles of Tregs in the pneumonitic phase and fibrotic phase of RILI. The ultimate goal is to evaluate whether Tregs hold promise as a potential target for immunotherapeutic interventions for RILI.

## RILI: A Disturbed Balance Between Inflammation and Tissue Repair

RILI can be generally divided into two distinct, yet tightly connected phases. One is an acute pneumonitic phase, termed RP, which manifests with dyspnea, non-productive cough, and fever within the first 6 months of radiotherapy (1). The second is RPF, a late, irreversible phase (>1 year following therapy) characterized by fibroblast activation, extracellular matrix (ECM) accumulation, and aberrant tissue remodeling (1). Patients with RPF may present with symptoms including dyspnea and respiratory insufficiency (24).

Radiation-induced damage to the resident cells (e.g., epithelial and endothelial cells) triggers a cascade of molecular events, including reactive oxygen species production and DNA damage, which results in an early immune response aimed at initiating tissue repair (20). The subsequent release of damage-associated molecular patterns (DAMPs) induces secretion of pro-inflammatory mediators such as interleukin (IL)−1 and tumor necrosis factor-α (TNF-α) through the activation of the nuclear factor kappa-B (NF-κB) signaling pathway (25), allowing activation of resident immune cells and recruitment of inflammatory cells (26), which, in turn, amplifies the ongoing inflammatory response (2, 26). Notably, accumulating evidence has pointed to the involvement of lymphocytes in radiation-induced early lung inflammation (17, 22, 27). For example, Cappuccini et al. described elevated levels of Th17-associated cytokines (e.g., IL-17 and IL-23) in the bronchoalveolar lavage fluid of irradiated mice at 21 days post thorax irradiation with 15 Gy (22), suggesting the contribution of pro-inflammatory Th17 responses to the development of RILI. Current treatment options for RP primarily focus on the control of excessive inflammatory responses with glucocorticoids, potent anti-inflammatory agents that are known to inhibit the expression of pro-inflammatory cytokines and abrogate the activity of NF- κB (28, 29).

In some cases, RP resolves after treatment with corticosteroids; in other cases, it evolves into an uncontrolled, fibrotic process, namely RPF. In contrast to the normal wound healing process, which involves a series of finely orchestrated biological events, RPF enters a “never-ending spiral,” in which tissue hypoxia, epithelial-mesenchymal transition (EMT), and fibroblast activation ultimately lead to the destruction of the normal lung architecture (2, 18, 30, 31). Transforming growth factor-β (TGF-β) has been recognized as a key signaling molecule in the fibrotic process (26), which stimulates fibroblast proliferation, EMT, and ECM production (26, 32). Furthermore, recent research has deepened our understanding of how lymphocytes - and, in particular, Tregs - are involved in the fibrotic process (16–23, 33). We speculate that a disturbed balance between inflammation and tissue regeneration is a central issue in RPF and that the interaction among Tregs, other infiltrating T cells, epithelial cells, and fibrocytes is instrumental in shifting the local environment toward one favoring fibrosis.

## Tregs and Immunoregulatory Cytokines

Two major subgroups of Tregs have been defined as follows, based on their differential developmental origins: (I) natural Tregs arising in the thymus (34) (II) inducible Tregs which can be induced from naïve CD4^+^ T cells by antigen exposure and cytokines such as TGF-β in the periphery (34–36). Mouse thymus-derived natural Tregs are characterized by their high expression of CD25 and constitutive expression of transcription factor forkhead box P3 (FoxP3) (37). In contrast to mouse Foxp3^+^ Tregs, human Foxp3^+^ T cells are more heterogeneous in function (38). For example, human naïve CD4^+^ T cells can transiently express Foxp3 after *in vitro* T-cell receptor stimulation without possessing suppressive functions (39–42). Therefore, Foxp3 *per se* may not represent a sufficient marker for functional Tregs, particularly in humans. In addition, Foxp3 is expressed at low levels or transiently after activation in some subsets of human Tregs (43, 44). Thus, identification of human Tregs is even more complex. At present, additional markers, such as CD127 and CD45RA, are also used to delineate Tregs in humans (45, 46).

Tregs in mice have been shown to exert suppression by cell-cell contact and humoral mechanisms with involvement of a variety of molecules, including surface molecules (e.g., cytotoxic T lymphocyte antigen-4 (CTLA-4), CD39, and CD73), immunoregulatory cytokines (e.g., TGF-β, IL-10, and IL-35), and secreted molecules (e.g., granzyme) (47). Several potential mechanisms of suppression by human Tregs have also been proposed, based on *in vitro* suppression assays, including cell-contact dependent mechanisms (e.g., Fas/FasL-mediated apoptosis of CD8^+^ responder cells and lysis of target cells through a perforin/granzyme-dependent pathway) and humoral factor-mediated mechanisms (e.g., secretion of inhibitory cytokines such as IL-10) (48–51). Notably, the mechanisms of human Treg-mediated suppression are similar, but not identical to, mechanisms of regulation by mouse Tregs. For example, IL-35 has been reported to be constitutively expressed by mouse Tregs and contribute to the suppressive activity of Tregs (52), whereas humans Tregs do not constitutively express IL-35 (53). In relations to the lungs, *in vivo* mouse studies have demonstrated IL-10 mediated Treg effects in allergic conditions (54, 55). In acute lung injury (ALI), Tregs were found to promote the resolution of inflammation through modulation of the alveolar inflammatory milieu in a mouse model of ALI (56). Furthermore, inhibition of TGF-β with neutralizing antibodies abrogated Treg-mediated resolution of inflammation in ALI (56), indicating a potential contribution of TGF-β-dependent mechanisms in the suppressive effects of Tregs. Additionally, recent observations demonstrate that mouse CD4^+^Foxp3^+^ Tregs in the lungs can also express the IL-33 receptor *suppression of tumorigenicity 2* (ST2) (57, 58). IL-33 is an alarmin cytokine that can be released from damaged epithelial cells to alert the immune system after tissue injury (59). Intriguingly, after exposure to IL-33, mouse Tregs were found to increase the expression levels of GATA-3 as well as ST2 and secrete Th2 cytokines (57). Moreover, it has been reported that mouse ST2^+^ Tregs secrete high levels of amphiregulin, IL-10, and TGF-β *in vitro* in the presence of IL-33 (58, 60). Thus, IL-33-responsive Tregs may have a role in tissue repair and fibrotic responses in the lungs, which needs to be confirmed in further studies.

## Tregs in Radiation-Induced Pneumonitis

As stated above, Tregs have been implicated in the resolution of inflammation in ALI and allergic conditions, and one can speculate that Tregs may also be beneficial during the pneumonitic phase of RILI in that they dampen excessive pro-inflammatory responses, thereby ameliorating inflammation-associated tissue damage.

Supporting this hypothesis, Wirsdorfer et al. found that thorax irradiation caused a transient accumulation of Tregs in irradiated lungs at 21 days after irradiation. Moreover, the accumulation of Tregs was associated with increased surface expression of immunoregulatory molecules (e.g., CTLA-4, CD73) on CD4^+^ T cells (17). It is tempting to speculate that Tregs play a protective role in the pneumonitic phase via their suppressive action on pro-inflammatory T cells, such as Th17 cells (22, 61, 62). Another murine study from Liu et al. demonstrated a transient increase in the number of Tregs expressing CTLA-4 in hilar lymph nodes and the spleen at 7 days after the silica exposure (12). Further, depletion of Tregs by intraperitoneal injection of anti-CD25 mAb 1 day before the silica exposure and every 7 days thereafter led to enhanced early lung inflammation with significantly increased infiltration of neutrophils at 7 days after the silica exposure (12), which lends further credence to the protective role of Tregs in the early inflammatory response to lung injury. Moreover, a recent clinical study reported that an imbalance of the Th17/Treg ratio toward Th17 cells was a predictor of RP in patients receiving thoracic radiotherapy (63).

There are several potential explanations for the accumulation of Tregs in irradiated lungs during the pneumonitic phase. First, the increased Treg fraction may be partially attributed to enhanced radioresistance and survival of Tregs compared with other lymphocyte subpopulations (10). Second, given the crucial role of TGF-β in the development of Tregs (34, 36), radiation-induced changes in the cytokine milieu (e.g., increased levels of TGF-β) may favor Treg accumulation in the lung tissue. However, it is noteworthy that radiation induces TGF-β production in a dose-dependent, time-dependent, and tissue-specific manner (64), and the precise role of TGF-β in the accumulation of Tregs in the lung tissue requires further exploration. Additionally, in irradiated macrophages, radiation-induced DNA damage results in potent induction of pro-inflammatory cytokines and chemokines (65). Tregs express a repertoire of inflammatory chemokines receptors (e.g., CCR2, CCR4, and CCR5) (66), suggesting that inflammatory chemokines may mediates the recruitment of Tregs at inflammatory sites through binding to their receptors on Tregs. For example, CCL2 has been shown to have a crucial role in the recruitment of Tregs to an inflamed site (67–69).

Current evidence indicates that Tregs can, via diverse mechanisms, dampen the inflammatory response after tissue injury and promote efficient repair of damaged tissue, which has been elegantly reviewed by Li et al. (70). Further studies are warranted to uncover the precise mechanisms whereby Tregs modulate inflammation in RILI.

## Tregs in Radiation-Induced Pulmonary Fibrosis

**Figure 1:**
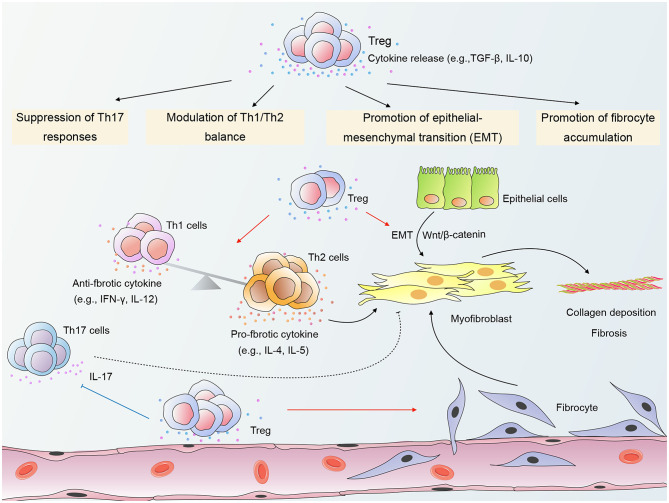
A schematic of mechanisms by which Regulatory T cells (Tregs) modulate radiation-induced pulmonary fibrosis (RPF). Tregs promote the accumulation of fibrocytes in irradiated lungs and β-catenin-mediated epithelial-mesenchymal transition (EMT) in epithelial cells. In addition, Tregs shift the Th1/Th2 cytokine balance toward Th2 dominance, thereby providing a cytokine milieu that favors fibrosis. Tregs may also modulate RPF through suppression of Th17 responses. The crosstalk among Tregs, other infiltrating T cells, epithelial cells, and fibrocytes contributes to the activation of myofibroblasts and collagen deposition, ultimately leading to the destruction of the normal lung architecture. “→”: transform or promote; “⊥”: inhibit; “- - -”: may play a role.

Current evidence indicates that Tregs exert a pro-fibrotic role in RPF (18, 21). Wirsdörfer et al. demonstrated in a mouse model that CD4^+^ FoxP3^+^ Tregs accumulated in irradiated lungs during the pneumonitic and fibrotic phase. Moreover, Tregs generally expressed CD73 (16). Of further interest are the findings by Xiong et al. demonstrating that long-term (6 months) depletion of Tregs by intraperitoneal injection of anti-CD25 mAb 2 h after thorax irradiation and every 7 days therefore effectively attenuated RPF in mice (18, 21). Notably, in these studies, the ablation of Tregs covered both the early phase and the late chronic phase. Therefore, it is plausible to speculate that Tregs may predominantly play a pro-fibrotic role in RILI. Tregs have been shown to contribute to RPF through several mechanisms, including promotion of fibrocyte accumulation, promotion of EMT, modulation of Th1/Th2 balance, and suppression of Th17 responses (shown in Figure 1) (18, 21).

### Promotion of Fibrocyte Accumulation

Fibrocytes are bone marrow-derived mesenchymal stem cells that can differentiate into myofibroblasts (71). The accumulation and differentiation of fibrocytes are key processes with massive implications for the pathogenesis of fibroproliferative diseases (72). Regarding RPF, Xiong et al. reported an increased number of fibrocytes in the lung tissue of mice during both early and chronic phase of RILI (21). Interestingly, Treg depletion reduced fibrocyte accumulation and attenuated lung fibrosis (21), indicating that Tregs may contribute to RPF by promoting fibrocyte accumulation in irradiated lungs.

Although the underlying mechanisms have not yet been clarified, Tregs may interact with fibrocytes through secretion of TGF-β, which is known to promote the proliferation, differentiation, and collagen production of fibrocytes (71, 73, 74). Additionally, the differentiation of Gr1^+^ monocytes into fibrocytes has been demonstrated to be largely under the control of CD4^+^ T cells (75). As a critical subset of CD4^+^ T cells, Tregs may also have a role in the development of fibrocytes. The interplay between Tregs and fibrocytes in RILI warrants further investigations.

### Promotion of Emt in Epithelial Cells

EMT is a process involving the transdifferentiation of epithelial cells with a progressive loss of apicobasal polarity and cell-cell contacts, as well as an acquisition of a mesenchymal phenotype (32). Growing evidence has demonstrated a key role of EMT in the pathogenesis of RPF (18, 76). Intriguingly, Treg depletion suppressed EMT and substantially attenuated pulmonary fibrosis in irradiated mice (18), revealing a detrimental role for Tregs in RPF through the promotion of EMT.

Furthermore, a study by Boveda-Ruiz et al. has shed light on the interaction between Tregs and epithelial cells, demonstrating that the co-culture of lung epithelial cells with Tregs resulted in increased protein and mRNA expression of TGF-β1 in epithelial cells (13). Thus, stimulation of TGF-β secretion from epithelial cells is a possible mechanism whereby Tregs accelerate RPF. Xiong et al. co-cultured irradiated lung epithelial cells with Tregs and found that Tregs promoted the EMT process and β-catenin expression in epithelial cells (18). Moreover, the EMT-promoting effect of Tregs was partially impaired by β-catenin silencing *in vitro* (18), suggesting that β-catenin-mediated EMT is a potential mechanism whereby Tregs modulate RPF.

### Modulation of Th1/Th2 Balance

Another possible mechanism whereby Tregs accelerate RPF is by shifting the Th1/Th2 cytokine balance toward Th2 dominance. Th2 signature cytokines such as IL-4 have been recognized as potent pro-fibrotic mediators due to their capacity to promote fibroblast activation and collagen production. In contrast to Th2 cytokines, Th1 signature cytokines such as interferon-γ (IFN-γ) exert suppressive effects on fibroblasts and reduce excessive collagen production (77). Therefore, Th2-dominant cytokine milieu may play a critical role in driving fibrosis.

In a mouse model of RPF, Treg depletion skewed the balance toward Th1 dominance with a cytokine profile of increased IFN-γ and IL-12 and decreased IL-4 and IL-5 (21). This observation is reminiscent of a study by Liu et al., demonstrating that Treg depletion in a mouse model of silica-induced lung fibrosis resulted in increased levels of Th1 cytokines and decreased Th2 cytokines (7). One possible mechanism whereby Tregs modulate the Th1/Th2 balance is via promoting the induction of alternatively activated (M2) macrophages (78), which are known to participate in the promotion of Th2 responses and tissue remodeling (79). Additionally, it has been demonstrated that Tregs can undergo molecular differentiation by modifying their expression of Th lineage-specific transcription factors such as T-box expressed in T cells (T-bet) and interferon regulatory factor-4 (IRF-4), thereby enabling them to specifically control Th1 or Th2 responses (80, 81). Thus, in RPF, Tregs may undergo molecular specialization in response to the local milieu that enables them to suppress Th1 responses, which ultimately drives a shift toward a Th2-dominant cytokine milieu.

### Suppression of Th17 Responses

To date, the contribution of Th17 cells to fibrotic disorders remains obscure. Animal studies revealed that antibody-mediated neutralization of IL-17A, a hallmark cytokine for Th17 cells, attenuated fibrosis in several damage-associated pulmonary diseases (82, 83). Moreover, in murine models of bleomycin-induced lung injury, IL-17 receptor A- or IL-17A-deficient mice displayed substantially reduced pulmonary fibrosis in contrast to wild-type mice (83, 84). By contrast, Lo Re et al. demonstrated that IL-17A was dispensable for the fibrotic response and that silica-induced pulmonary fibrosis was not attenuated upon treatment with anti-IL-17A antibody or in IL-17R- deficient mice (85).

Evidence suggests that the imbalance of Th17/Treg is a possible mechanism whereby Tregs modulate RPF. According to a preclinical study by Xiong et al. Treg depletion attenuated lung fibrosis, which was accompanied by a significant increase in the number of Th17 cells from month 3 to 6 after irradiation (21), suggesting that Tregs may accelerate RPF through suppression of Th17 responses and that Th17 cells may exert an anti-fibrotic function in RPF. By contrast, Paun et al. demonstrated that the IL-17A-deficient mice were protected from RPF, indicating a pathogenic role of Th17 cells in RPF (86).

Tregs have been shown to restrain Th17 responses in a signal transducers and activators of transcription 3 (Stat3)-dependent manner (87). Meanwhile, other studies have reported that Tregs can promote Th17 differentiation and augment IL-17A induction (88–90). These conflicting findings reflect the complexity of the interplay between Tregs and Th17 cells. The interaction between Tregs and Th17 cells in RPF requires further exploration.

## Therapeutic Implications and Future Directions

Notably, the interaction of Tregs with other immune cells (e.g., macrophages) and damaged resident cells (e.g., endothelial cells) may also impact the development of RILI. Tregs can profoundly regulate macrophage phenotype and function (79). For example, co-culture of human monocytes with Tregs steers monocyte differentiation toward M2 macrophages (78), which are known to participate in the resolution of inflammation and tissue remodeling (79). Additionally, macrophages co-cultured with Tregs produce increased levels of TGF-β and decreased levels of TNF-α in response to lipopolysaccharide (56). Thus, we speculate that the interaction between Tregs and macrophages may be involved in tissue repair and pulmonary fibrosis induced by radiation, which warrants further investigation. Additionally, the interaction between Tregs and endothelial cells (ECs) has attracted special attention due to its potential involvement in the regulation of inflammation (91–93). Human umbilical vein endothelial cells co-cultured with Tregs show a diminished capacity to respond to lipopolysaccharide in terms of adhesion molecule expression and pro-inflammatory cytokine production (e.g., monocyte chemoattractant protein-1 and IL-6), suggesting a protective role of Tregs during inflammation (93). In turn, ECs can also affect Treg function, as suggested by Bedke et al., who showed that activated murine lung ECs stimulated the release of IL-10 and TGF-β from Tregs and triggered upregulation of programmed death-1 (PD-1) by Tregs (91). Further investigation on the role of EC–Treg interaction in RILI may provide new insights into the pathogenesis of RILI.

Recognition of the critical role of Tregs in RPF has prompted interest in targeting Tregs to prevent or treat RPF. The observation that Treg depletion attenuated RPF is of particular interest in the era of cancer immunotherapy, as Treg depletion has been shown to have potential for reversing immunosuppression when combined with radiotherapy (11). Nevertheless, targeting Tregs for the treatment of RPF is still limited to preclinical models, and its translation into clinical application remains challenging. One key challenge is to determine the optimal timing for removal of Tregs, given that Tregs may have a requisite role in protecting normal tissue against excessive inflammation-induced damage during the early stage of RILI.

Recently, the finding that CD73 potentiated RPF has prompted interest in targeting CD73 to limit radiation-induced lung toxicity (16). Up to now, although CD73^+^CD4^+^Foxp3^+^ Tregs have been shown to accumulate in irradiated lungs during the pneumonitic and fibrotic phase (16), the role of CD73^+^CD4^+^Foxp3^+^ Tregs in radiation-induced pneumopathy is poorly defined. We speculate that CD73-targeted therapies may limit potential pro-fibrotic actions of Tregs in pulmonary fibrosis induced by radiation. Moreover, given the crucial role of CD73 expression on Tregs in the suppression of antitumor immunity (94, 95), it is intriguing to speculate that CD73-targeted therapy may provide therapeutic benefits by promoting antitumor immunity and reducing late radiation toxicity. Additionally, emerging evidence highlights a novel contribution of the IL-33/ST2 pathway to fibrotic disorders (59). Regarding pulmonary fibrosis, Li et al. reported that bleomycin-induced lung fibrosis was attenuated in ST2-deficient mice or upon treatment with anti–IL-33 antibody (96). Moreover, exogenous administration of mature recombinant IL-33 exacerbated bleomycin-induced fibrosis in mice (96), indicating a critical role of IL-33 in the fibrotic response to lung injury. To date, the mechanisms, especially involving Tregs, whereby IL-33 contributes to pulmonary fibrotic disorders remain underexplored, although some studies have demonstrated strong immunosuppressive properties and Th2-like character of IL-33 activated Tregs *in vitro* (57, 58, 60). A recent study in mice has revealed a protective role for IL-33 in ALI, demonstrating that IL-33-mediated control of inflammation involves the stimulation of IL-13 secretion by ST2^+^ Tregs, which reduces the infiltration of inflammatory monocytes and local inflammatory cytokines, such as IL-6 and granulocyte-colony stimulating factor (G-CSF) (97). It is possible to envision that the sustained increase in epithelial-derived IL-33 and chronic activation of ST2^+^ Tregs triggered by irradiation may eventually shift the local environment toward one favoring fibrosis. Further investigation on the role of IL-33 activated Tregs in radiation-induced pneumopathy is warranted and may provide insight into the therapeutic potential of IL-33/ST2 in RILI.

## Concluding Remarks

Accumulating evidence has demonstrated a profound, yet complex role of Tregs in RILI. During the pneumonitic phase, Tregs may play a role in counterbalancing exaggerated pro-inflammatory responses and preventing the worsening of radiation-induced injury. On the other hand, emerging data point to the crucial involvement of Tregs in RPF. The role of Tregs in RPF is likely to be multi-factorial – related to promotion of fibrocyte accumulation, promotion of EMT, and modulation of Th1/Th2 balance.

Although growing interest has been focused on the role of Tregs in RILI and that Treg depletion has been shown to attenuate RILI in mice, from a therapeutic viewpoint, this field is still in its infancy. Further studies that probe into unanswered questions, such as the interaction of Tregs with other cells in RILI, represent the next steps forward in developing effective therapeutic strategies. Given the critical role of Tregs in both tumor-induced immune suppression and radiation-induced fibrotic response, we envision that Tregs represent a promising target for future treatment options. A deeper understanding of the mechanisms whereby Tregs modulate RILI will offer new avenues for the efficient management of RILI.

## Author Contributions

TG and LZ drafted the manuscript. JN, YZ, and LY reviewed and edited the manuscript. XY and ZZ conceived the topic and revised the manuscript. All authors read and approved the final manuscript.

## Conflict of Interest

The authors declare that the research was conducted in the absence of any commercial or financial relationships that could be construed as a potential conflict of interest.

## Abbreviations

ALI: Acute lung injury
CTLA-4: Cytotoxic T lymphocyte antigen-4
DAMPs: Damage-associated molecular patterns
ECs: Endothelial cells
ECM: Extracellular matrix
EMT: Epithelial-mesenchymal transition
FoxP3: Forkhead box P3
G-CSF: Granulocyte-colony stimulating factor
IFN-γ: Interferon-γ
IL: Interleukin
IRF-4: Interferon regulatory factor-4
M2 macrophage: Alternatively activated macrophage NF-κB, Nuclear factor kappa-B
PD-1: Programmed death-1
RILI: Radiation-induced lung injury
RP: Radiation-induced pneumonitis
RPF: Radiation-induced pulmonary fibrosis
ST2: Suppression of tumorigenicity 2
Stat3: Signal transducers and activators of transcription 3
T-bet: T-box expressed in T cells
TGF-β: Transforming growth factor-β
TNF-α: Tumor necrosis factor-α
Tregs: Regulatory T cells.
